# Diagnostic Performance of the α-Synuclein Seed Amplification Assay for Dementia With Lewy Bodies: A Comparison Across 4 Laboratories

**DOI:** 10.1212/WNL.0000000000214614

**Published:** 2026-01-28

**Authors:** Rakesh Kumar, Stephanie Gravett, Vesna Jelic, Johannes Lange, Linn Oftedal, Arianna Ciullini, Merve Begüm Bacınoğlu, Chiara Maria Giulia De Luca, Lola Hamied, Catherine Birck, Frederic Blanc, Patty L. Hoede, Afina W. Lemstra, Maria Camila Gonzalez, Dag Aarsland, Charlotte E. Teunissen, Olivier Bousiges, Fabio Moda, Jodi Maple-Grødem, Axel Abelein, Daniel Ferreira

**Affiliations:** 1 Department of Neurobiology Care Sciences and Society, Center for Alzheimer Research, Division of Clinical Geriatrics, Karolinska Institutet, Huddinge, Sweden;; 2 Department of Medicine Huddinge, Karolinska Institutet, Sweden;; 3 Cognitive Disorders Clinic, Theme Inflammation and Aging, Karolinska University Hospital-Huddinge, Stockholm, Sweden;; 4 Centre for Movement Disorders, Stavanger University Hospital, Norway;; 5 Department of Chemistry, Bioscience and Environmental Engineering, University of Stavanger, Norway;; 6 Unit of Laboratory Medicine, Laboratory of Clinical Pathology, IRCCS Neurologic Institute Carlo Besta, Milan, Italy;; 7 Department of Neurology 5 - Neuropathology, Fondazione IRCCS Istituto Neurologico Carlo Besta, Milan, Italy;; 8 Laboratory of Biochemistry and Molecular Biology, University Hospital of Strasbourg, Hautepierre Hospital, France;; 9 Plateforme de Biologie Structurale Intégrée (BSI-FRISBI), CBI-IGBMC, CNRS UMR 7104, Inserm U1258, University of Strasbourg, Illkirch, France;; 10 University Hospitals of Strasbourg, CM2R (Research and Resources Memory Center), Day Hospitals, Service of Gerontology Mobile-Neuro-Psy-Research, GeRMINED Department, France;; 11 University of Strasbourg and CNRS, ICube Laboratory UMR 7357 and FMTS (Fédération de Médecine Translationnelle de Strasbourg), IMIS Team, France;; 12 Neurochemistry Laboratory, Department of Laboratory Medicine, Amsterdam UMC, Vrije Universiteit Amsterdam, Amsterdam Neuroscience, the Netherlands;; 13 Alzheimer Center, Department of Neurology, Amsterdam UMC, Vrije Universiteit Amsterdam, Amsterdam Neuroscience, the Netherlands;; 14 Centre for Age-Related Medicine, Stavanger University Hospital, Norway;; 15 Department of Psychological Medicine, Institute of Psychiatry, Psychology and Neuroscience, King's College London, United Kingdom;; 16 Department of Medical Biotechnology and Translational Medicine, University of Milan, Italy; and; 17 Facultad de Ciencias de la Salud, Universidad Fernando Pessoa Canarias, Las Palmas, Spain.

## Abstract

**Background and Objectives:**

The α-synuclein (α-syn) seed amplification assay (SAA) has shown promising results for diagnosing dementia with Lewy bodies (DLB) using CSF samples. A barrier to implementing α-syn SAA clinically is the use of different protocols for the assay. It is unknown how different protocols perform in comparison with each other. We compared the performance of α-syn SAA across 4 laboratories using CSF samples of patients with DLB.

**Methods:**

This was a retrospective cross-sectional study that included data from 4 different European laboratories. We included probable patients with DLB with a positive dopamine transporter (DaT)-SCAN and known amyloid-β status who had mild-to-moderate dementia, along with age-matched and sex-matched controls. The α-syn SAA was run across 4 laboratories using different protocols varying α-syn concentration and plate reader settings. CSF samples were provided by a fifth independent laboratory, which also performed statistical and result analyses.

**Results:**

We included 20 patients with DLB (mean age 67 ± 6 years, 60% male) and 10 controls (mean age 67 ± 2 years, 70% male). Neuropathologic confirmation was available for 2 patients with DLB. On average, the 4 laboratories achieved 78.8% sensitivity (minimum 55%, maximum 100%), 77.5% specificity (minimum 60%, maximum 100%), and 78.5% accuracy (minimum 57%, maximum 100%) for discriminating DLB from controls, but our findings show that diagnostic performance of SAA varied across laboratories: Lab A achieved 100% sensitivity (CI 84%–100%) and 100% specificity (CI 72%–100%); Lab B achieved 85% sensitivity (CI 64%–95%) and 90% specificity (CI 59%–99%); Lab C achieved 55% sensitivity (CI 34%–74%) and 60% specificity (CI 31%–83%); and Lab D achieved 75% sensitivity (CI 53%–89%) and 60% specificity (CI 31%–83%). In general, SAA results showed numerically lower sensitivity in β-amyloid (Aβ)–positive patients with DLB (70%) compared with Aβ-negative patients with DLB (87.5%) (nonstatistically significant). A fair agreement of SAA results was obtained across the 4 laboratories (average κ = 0.246).

**Discussion:**

This study highlights challenges for the reproducibility of α-syn SAA results across different protocols applied by different laboratories. This finding, together with the methodological variability reported across laboratories, may challenge the clinical implementation of the α-syn SAA. This study provides relevant support for initiating harmonization and standardization of SAA protocols to move the field toward the clinical implementation of SAAs for the biomarker-based diagnosis of DLB.

**Classification of Evidence:**

This study provides Class III evidence of variations in the accuracy of CSF α-syn SAA across 4 separate laboratories in distinguishing patients with DLB from healthy controls.

## Introduction

The clinical diagnosis of dementia with Lewy bodies (DLB) is complex and challenging because of overlapping symptoms with Alzheimer disease (AD) and other neurodegenerative disorders.^1^ The diagnosis is based on core clinical features, cognitive tests, and various neuroimaging biomarkers, among which dopamine transporter imaging (DaT-SCAN) is an indicative biomarker of DLB.^2^

Neuronal α-synuclein (α-syn) misfolding and aggregation are characteristic of synucleinopathies such as DLB and Parkinson disease (PD).^3,4^ In addition, around 50% of patients with DLB are positive for β-amyloid (Aβ) biomarkers.^5^ α-Syn is present in various biofluids such as CSF and blood.^6^ The quantification of α-syn biomarkers in these biofluids using ELISA or Western blot has provided inconclusive results, limiting the diagnostic utility of these 2 methods for α-syn.^7,8^

By contrast, seed amplification assay (SAA) has been reported to be an ultrasensitive method that amplifies tiny amounts of misfolded proteins in biofluids and tissue.^9^ In synucleinopathies, SAA shows sensitivity values ranging from 81% to 100% when differentiating patients with DLB from healthy controls, cognitive unimpaired individuals, or people with other neurologic disorders and specificity values ranging from 91% to 100%.^10^ Specifically in DLB, α-syn SAA shows sensitivity and specificity values of 94% and 96%, respectively, for differentiating patients with DLB from controls.^11^

Although the α-syn SAA has shown a very high diagnostic performance for DLB (and PD),^10^ its clinical implementation is hampered by several factors, such as lack of standardization and reproducibility of the SAA across different laboratories, lack of consensus on cut points to define a positive biomarker result, and lack of quantification of α-syn seeds in biofluids.^12^ Another major unmet need for the clinical implementation of the α-syn SAA is cross-validation in multiple laboratories. A previous study compared 2 different α-syn SAA protocols across 2 laboratories in patients with PD. The results showed 92% concordance of the assay, with a sensitivity of 96% and a specificity of 82%.^13^ Another study compared the α-syn SAA across 3 laboratories in 30 patients with PD, 30 controls, and 20 patients with scans without evidence of dopaminergic deficit.^14^ That study showed sensitivity values of 86%–96% and specificity values of 97%–100% for PD.^14^ A recent study comparing the α-syn SAA across 4 laboratories included 14 patients with PD, 4 patients with DLB, and 20 controls.^15^ They showed high agreement in qualitative SAA results, whereas quantitative analysis of interlaboratory agreement showed a fair agreement across laboratories.^15^ However, the previous study was performed using only 2 different protocols applied across 4 laboratories, and the group of patients with DLB was small. Therefore, further cross-validation studies on α-syn SAAs are needed, which include multiple laboratories, test different protocols, and use α-syn monomer from different sources, particularly in patients with DLB, given that aggregation kinetics of α-syn SAA differ between DLB and PD.^16^

Our primary research aim was to compare the performance of the α-syn SAA across 4 European laboratories using CSF samples from the same patients with DLB (head-to-head). The specific aims were (1) to compare sensitivity, specificity, and accuracy values of the α-syn SAA across laboratories, (2) to study the effect of Aβ status on SAA results in patients with DLB, (3) to analyze the agreement of SAA results across laboratories, and (4) to assess differences and similarities of the SAA protocol across laboratories.

## Methods

### Participants

We included patients with DLB and cognitively unimpaired age-matched controls from the Find-DLB project.^17^ All patients with DLB were required to have a positive DaT-SCAN and mild-to-moderate dementia. For patients with DLB, CSF samples were required to be either Aβ positive (Aβ^+^) or Aβ negative (Aβ^−^). For Aβ biomarker interpretation, CSF Aβ 42/40 values were compared with reference values validated in the clinical laboratory on site. One patient with DLB had a neuropathologic confirmation of DLB, and another patient had a neuropathologic confirmation of DLB with concomitant AD.

Clinical diagnosis in patients and controls was based on comprehensive assessment at the Cognitive Clinic at Karolinska University Hospital Huddinge (Stockholm, Sweden). Patients with DLB were diagnosed using the international consensus criteria for probable DLB.^2^ For core clinical features, parkinsonism was assessed by the clinicians, whereas visual hallucinations (VHs), probable REM sleep behavior disorder (RBD), and cognitive fluctuations were reported by patients, caregivers, or their next of kin. Global cognitive performance was tested using the Montreal Cognitive Assessment and/or Mini-Mental State Examination (MMSE). If clinically relevant, patients underwent a full neuropsychological protocol described elsewhere.^17^ Controls underwent the same assessment procedures as patients, and after extensive neuropsychological testing, no cognitive impairment or neurologic disorders were detected.

### Standard Protocol Approvals, Registrations, and Participant Consents

All participants gave consent, and the study was approved by the regional ethics committee in Stockholm with diary numbers 2013/2169-31, 2019–05978, and 2022-01251-02, including analysis of retrospective clinical data.

### CSF Samples and SAA

CSF samples were obtained from the Karolinska Institutet GEDOC Biobank (Stockholm, Sweden) and stored at Karolinska Institutet (Sweden). Next, samples were sent to 4 laboratories in Europe for SAA analysis (denoted as Lab A, Lab B, Lab C, and Lab D in this article). The 4 laboratories were blinded to any information about the samples. Each laboratory ran the SAA locally and sent the results back to Karolinska Institutet for independent statistical analyses.

α-Syn protein was expressed and purified in *E. coli* for Lab A. The detailed protocol is described in the eMethod. Labs B and C used α-syn from rPeptide (Bogart, GA), and Lab D obtained α-syn protein from Amprion (San Diego, CA).

Each laboratory ran their own SAA protocol and applied their local cutoff criteria for determining positive/negative SAA results. Detailed protocols and cutoff criteria are described in the eMethod.

### Statistical Analysis

We report the SAA result as positive, negative, or inconclusive. Sensitivity, specificity, and accuracy values were calculated. We plot maximum and median Thioflavin T (ThT) fluorescence of all replicates for each patient and control. The *t* test (Mann-Whitney) was performed for demographic and clinical variables, both for comparing patients with DLB with controls and Aβ^+^ patients with DLB with Aβ^−^ patients with DLB. Mann-Whitney and Wilcoxon matched-pair signed rank tests were also used for interlab comparison of sensitivity, specificity, and accuracy values. Cohen κ was used to assess agreement of SAA results and interpreted with the scale provided in the supplementary material.^18^ Analyses and plotting were performed with Prism 10.0.

### Data Availability

SAA data and results analysis information are available from corresponding author on reasonable request.

## Results

### Demographic and Clinical Characteristics

Table 1 provides the main characteristics of the cohort, which includes 20 patients with DLB and 10 cognitively unimpaired age-matched and sex-matched controls. By study design, 10 patients with DLB were Aβ^+^ and 10 patients with DLB were Aβ^−^. Most of the patients with DLB showed parkinsonism (90%), 60% showed VHs, and 59% showed cognitive fluctuations. The least frequent clinical feature in this cohort was RBD, which was reported in 50% of patients with DLB. Aβ^+^ patients with DLB did not differ significantly from Aβ^−^ patients with DLB in age, sex distribution, years of education, total MMSE score, or frequency of clinical features. Core clinical features are also provided in Table 2 for each individual patient with DLB.

**Table 1:** Demographic and Clinical Characteristics of Participants

	Controls (n = 10)	Patients with DLB (n = 20)	*p* Value	DLB Aβ^+^ (n = 10)	DLB Aβ^−^ (n = 10)	*p* Value
Age, y, mean ± SD	67 ± 2	67 ± 6	0.7049	69 ± 5	66 ± 6	0.315
Sex, % male	70	60	0.7020	60	60	>0.999
Education, y, mean ± SD	14.65 ± 2.98	11.52 ± 3.11 (n = 19)	0.0139	11.40 ± 2.45	11.66 ± 3.87 (n = 9)	0.759
MMSE score, mean ± SD	26 ± 3 (n = 7)	23.76 ± 3.70 (n = 17)	0.1396	24.50 ± 3.25 (n = 8)	23.11 ± 4.13 (n = 9)	0.587
Parkinsonism, %	NA	90		80	100	0.473
Cognitive fluctuations, %	NA	59 (n = 17)		57 (n = 7)	60	>0.999
VH, %	NA	60		60	60	>0.999
Probable RBD, %	NA	50 (n = 18)		50	50 (n = 8)	>0.999
DaT^+^, %	NA	100		100	100	>0.999
DaT^+^ Aβ^+^, %	NA	50		100	0	<0.0001
DaT^+^ Aβ^−^, %	NA	50		0	100	<0.0001

Abbreviations: Aβ = β-amyloid; DaT = dopamine transporter; MMSE = Mini-Mental State Examination; NA = not available; RBD = REM sleep behavior disorder; VH = visual hallucination.

The ^+^ sign aside DaT and Aβ reflects that the biomarker is positive while the ^−^ sign reflects that the biomarker is negative.

**Table 2:** Performance of SAA Across 4 Laboratories in Patients With DLB and Controls

Group	Participant	Lab A		Lab B		Lab C		Lab D		Core clinical features					DaT-SCAN
		Positive/4 or 8	SAA result	Positive/3 or 6	SAA result	Positive/3	SAA result	Positive/3	SAA result	Parkinsonism	Cognitive fluctuations	VH	RBD	Sum of core clinical features	
Aβ negative	1	4	+	4/6	+	0	−	2	+	+	+	−	−	2	+
	2	4	+	3	+	2	+	2	+	+	−	+		2	+
	3	4	+	3	+	3	+	2	+	+	+	+	−	3	+
	4	4	+	2	+	1	?	1	?	+	+	−	+	3	+
	5	4	+	3	+	2	+	3	+	+	−	+	+	3	+
	6	4	+	3	+	1	?	3	+	+	−	−		1	+
	7	4	+	3	+	2	+	3	+	+	+	+	+	4	+
	8	4	+	2	+	3	+	3	+	+	+	+	−	3	+
	9	4	+	3	+	1	?	3	+	+	+	+	+	4	+
	10	3	+	3	+	2	+	3	+	+	−	−	−	1	+
Aβ positive	11	4	+	4/6	+	0	−	3	+	+		−	−	1	+
	12	4	+	3	+	2	+	3	+	+	+	+	+	4	+
	13	3	+	3	+	2	+	3	+	+	−	−	−	1	+
	14	3	+	0	−	1	?	1	−	+		−	−	1	+
	15	4	+	3	+	3	+	2	+	+	+	+	−	3	+
	16	4	+	0	−	0	−	0	−	−	−	+	+	2	+
	17	4	+	3	+	2	+	3	+	+	+	+	−	3	+
	18	4	+	3	+	2	+	2^a^	?	+		−	+	2	+
	19	3	+	0	−	0	−	1	−	+	+	+	+	4	+
	20	4	+	3	+	1	?	3	+	−	−	+	+	2	+
Controls	21	0	−	0	−	2	+	0	−						
	22	0	−	1/6	−	0	−	2^a^	?						
	23	0	−	1/6	−	1	?	0	−						
	24	0	−	2/6	+	1	?	1	?						
	25	0	−	0	−	1	?	0	−						
	26	0	−	0	−	0	−	1	−						
	27	0	−	1/6	−	0	−	3^a^	?						
	28	0	−	0	−	0	−	3	+						
	29	1/8	−	0	−	0	−	0	−						
	30	1/8	−	1/6	−	0	−	1	−						

Abbreviations: Aβ = β-amyloid; DLB = dementia with Lewy bodies; SAA = seed amplification assay.

Results are reported as positive (+), negative (−), or inconclusive (?). Blank is given for unknown. The table shows the number of replicates that are positive out of the total number of replicates used in the assay. Labs A and B repeated the SAA for inconclusive cases, and therefore, the number of replicates for Lab A is 4 or 8 (for inconclusive cases in initial SAA results) and that for Lab B is 3 or 6. Patient 9 had a neuropathologic confirmation for Lewy body disease, and patient 18 had a neuropathologic confirmation of Lewy body disease with AD co-pathology. The 4 core clinical features of DLB are shown, along with the sum of all features for the patients with DLB. Core clinical features were not recorded for controls and for a few patients with DLB; therefore, cells in the table are blank.

a For these samples, the replicate aggregated before 48 hours and, therefore, it is considered inconclusive.

### Performance of SAA Across 4 Laboratories in Patients With DLB and Controls

The SAA results are summarized in Table 2 as positive, negative, or inconclusive. Inconclusive results were defined individually for each laboratory as explained in the Supplementary Material. SAA results are also shown in the Figure, which presents the maximum relative fluorescence intensity of each replicate.

**Figure:**
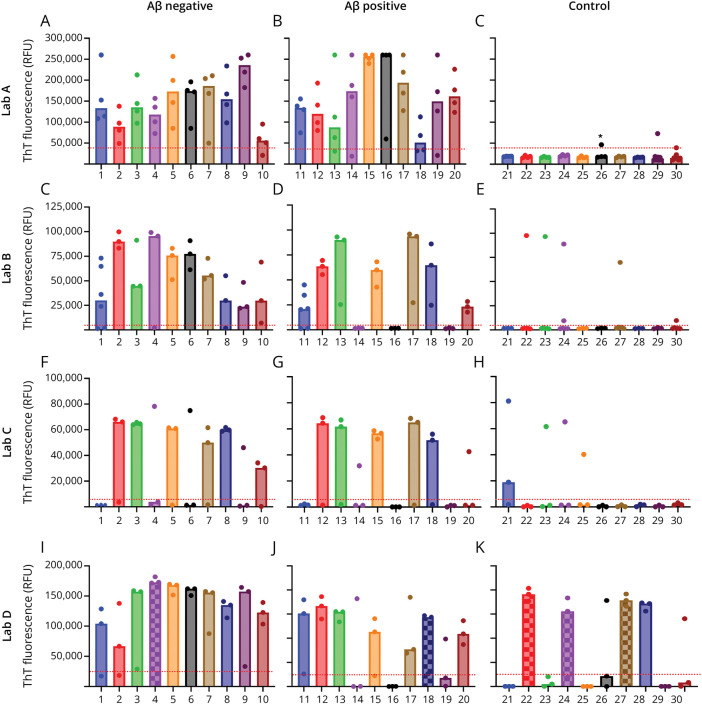
Bar Plot Showing the Median of the Maximum ThT Fluorescence Intensity Across 4 Laboratories for Patients With DLB and Controls Bar plot results are divided into 3 groups: Aβ-negative, Aβ-positive, and control groups. The cutoff fluorescence values for positive results are shown as dotted red lines. If 2 or more replicates cross the cutoff fluorescence, the sample is marked as SAA positive from Labs A to C. For Lab D, SAA is positive when the median fluorescence of 3 replicates crosses the cutoff fluorescence. A squared bar in Lab D shows inconclusive results. For Lab A, the cutoff fluorescence was the average fluorescence of all wells in the first 10 hours of the assay plus 30 times SD. (*) One of the replicates is slightly above the cutoff value in the plot, but this sample is negative because the individual plate ThT fluorescence cutoff is lower than the average fluorescence of all 3 plates. This is explained in the eMethod. Data from Labs A and B include the repeated SAA results for inconclusive cases. Therefore, data from 8 replicates are shown for 2 cases in Lab A and 6 replicates for 7 cases in Lab B. Aβ = β-amyloid; ThT = Thioflavin T

The SAA results were analyzed at 3 levels as initial, actual, and final. Initial results take into account only conclusive results after the first SAA run. For actual SAA results, cases with inconclusive results after the first SAA run were also included in the analysis but considered failures for SAA. Labs A and B repeated the assay for inconclusive cases, whereas Labs C and D were unable to repeat the assay for inconclusive cases because of insufficient CSF volume available after the first SAA run. Final SAA results were obtained after repeating tests on inconclusive cases (Table 2).

### Sensitivity, Specificity, and Accuracy of the α-Syn SAA Across Laboratories

Table 3 provides a summary of sensitivity, specificity, and accuracy values for each laboratory.

**Table 3:** Summary of Sensitivity Specificity and Accuracy Metrics Across 4 Laboratories Reported as Initial, Actual, and Final Sensitivity, Specificity, and Accuracy Values

Laboratories	Inconclusive SAA results	Sensitivity (in %)			Specificity (in %)			Accuracy (in %)		
		Initial	Actual	Final	Initial	Actual	Final	Initial	Actual	Final
Lab A	2 cases (repeated)	**100** (CI 84–100) (20/20) PPV 100 (CI 84–100)	**100** (CI 84–100) (20/20) PPV 100 (CI 84–100)	**100** (CI 84–100) (20/20) PPV 100 (CI 84–100)	**100** (CI 68–100) (8/8) NPV 100 (CI 68–100)	**80** (CI 49–96) (8/10) NPV 100 (CI 68–100)	**100** (CI 72–100) (10/10) NPV 100 (CI 72–100)	**100** (CI 88–100) (28/28) PPV 100 (CI 88–100)	**93** (CI 79–99) (28/30) PPV 100 (CI 88–100)	**100** (CI 88–100) (30/30) PPV 100 (CI 88–100)
Lab B	7 cases (repeated)	**83** (CI 61–94) (15/18) PPV 100 (CI 80–100)	**75** (CI 53–89) (15/20) PPV 100 (CI 80–100)	**85** (CI 64–95) (17/20) PPV 100 (CI 82–100)	**100** (CI 57–100) (5/5) NPV 100 (CI 57–100)	**50** (CI 24–76) (5/10) NPV 100 (CI 57–100)	**90** (CI 59–99) (9/10) NPV 100 (CI 70–100)	**87** (CI 68–95) (20/23) PPV 100 (CI 84–100)	**67** (CI 49–81) (20/30) PPV 100 (CI 84–100)	**87** (CI 70–95) (26/30) PPV 100 (CI 87–100)
Lab C	8 cases (not repeated)	**73** (CI 48–89) (11/15) PPV 100 (CI 74–100)	**55** (CI 34–74) (11/20) PPV 100 (CI 74–100)	**55** (CI 34–74) (11/20) PPV 100 (CI 80–100)	**85** (CI 49–99) (6/7) NPV 100 (CI 61–100)	**60** (CI 31–83) (6/10) NPV 100 (CI 61–100)	**60** (CI 31–83) (6/10) NPV 100 (CI 61–100)	**77** (CI 57–90) (17/22) PPV 100 (CI 82–100)	**57** (CI 39–73) (17/30) PPV 100 (CI 82–100)	**57** (CI 39–73) (17/30) PPV 100 (CI 82–100)
Lab D	5 cases (not repeated)	**83** (CI 61–94) (15/18) PPV 100 (CI 80–100)	**75** (CI 53–89) (15/20) PPV 100 (CI 80–100)	**75** (CI 53–89) (15/20) PPV 100 (CI 80–100)	**85** (CI 49–99) (6/7) NPV 100 (CI 61–100)	**60** (CI 31–83) (6/10) NPV 100 (CI 61–100)	**60** (CI 31–83) (6/10) NPV 100 (CI 61–100)	**84** (CI 65–94) (21/25) PPV 100 (CI 85–100)	**70** (CI 52–83) (21/30) PPV 100 (CI 85–100)	**70** (CI 52–83) (21/30) PPV 100 (CI 85–100)
Significant pairwise comparisons		A > C (*p* = 0.026)	A > C (*p* = 0.003)	A > C (*p* = 0.003) B > C (*p* = 0.031)				A > C (*p* = 0.0124) A > D (*p* = 0.043)	A > B (*p* = 0.021) A > C (*p* = 0.007)	A > C (*p* = > 0.0002) A > D (*p* = 0.003) B > C (*p* = 0.003)

Abbreviations: NPV = negative predictive value; PPV = positive predictive value; SAA = seed amplification assay.

Initial sensitivity, specificity, and accuracy were calculated after the first run of SAA where inconclusive cases were excluded from the analysis. Actual sensitivity, specificity, and accuracy were calculated by considering inconclusive cases as failures (nonsuccess), with or without repeating samples for SAA. Final sensitivity, specificity, and accuracy were calculated after repeating SAA for inconclusive cases. Lab A had 2 inconclusive cases after initial SAA, Labs C and D did not repeat inconclusive cases, and therefore, final sensitivity, specificity and accuracy values are the same as actual sensitivity, specificity and accuracy. Numbers in bracket show the number of samples that tested positive or negative out of the total number of samples. Sensitivity, specificity, and accuracy values are reported as percentage (bold number), and in bracket, 95% CIs are shown in percentage. PPVs and NPVs are reported in percentage with 95% CIs. Pairwise comparison is shown in the last row for pairs, which showed significance in the statistical test.

#### Lab A

In the initial analysis of the conclusive SAA results (samples that resulted in either positive or negative α-syn SAA), all 20 patients with DLB had a positive α-syn SAA result, thus achieving 100% sensitivity, and all 8 controls had a negative α-syn SAA result, thus achieving 100% specificity. Two controls had an inconclusive SAA result. When considering these 2 inconclusive cases, the actual performance dropped to 8/10, thus yielding 80% specificity. To resolve the inconclusive cases, Lab A repeated the SAA for the 2 controls. The repeat analysis showed a negative α-syn SAA result for both cases, resulting in 100% specificity (10/10 after reanalysis). Therefore, the final diagnostic performance of α-syn SAA in Lab A was 100% sensitivity, 100% specificity, and 100% accuracy (30/30).

#### Lab B

Initial results showed a positive α-syn SAA in 15 of 18 patients (83% sensitivity) and a negative α-syn SAA in all 5 controls (100% specificity). When considering inconclusive results, the actual SAA performance was 15/20 for patients (75% sensitivity) and 5/10 for controls (50% specificity). The final SAA performance yielded 85% sensitivity (17/20 after reanalysis), 90% specificity (9/10 after reanalysis), and 87% accuracy (26/30).

#### Lab C

Initial results showed a positive α-syn SAA in 11 of 15 patients (73% sensitivity) and a negative α-syn SAA in 6 of 7 controls (85% specificity). When inconclusive cases were considered, the actual diagnostic performance dropped to 55% sensitivity (11/20) and 60% specificity (6/10), with an accuracy of 57% (17/30). The SAA could not be repeated for inconclusive cases, so actual performance reflects final performance.

#### Lab D

Initial results showed a positive α-syn SAA in 15 of 18 patients (83% sensitivity) and a negative α-syn SAA in 6 of 7 controls (85% specificity). When inconclusive cases were considered, the actual diagnostic performance decreased to 75% sensitivity (15/20) and 60% specificity (6/10), with an accuracy of 70% (21/30). The SAA assay was not repeated for inconclusive cases, so the actual performance reflects the final performance.

Table 3 presents pairwise statistical comparisons across laboratories. We found statistically significant differences for initial sensitivity (Lab A > Lab C), actual sensitivity (Lab A > Lab C), and final sensitivity (Labs A and B > Lab C). Nonstatistically significant differences were observed for specificity values. Statistically significant differences were observed for initial accuracy (Lab A > Labs C and D), actual accuracy (Lab A > Labs B and C), and final accuracy (Lab A > Labs C and D and Lab B > Lab C).

#### Overall Performance of the α-Syn SAA Across Laboratories

Based on final SAA results, the average sensitivity was 78.8% (minimum 55%, maximum 100%), specificity was 77.5% (minimum 60%, maximum 100%), and accuracy was 78.5% (minimum 57%, maximum 100%).

### Effect of Aβ Status on SAA Results in Patients With DLB

To assess whether Aβ influences the performance of α-syn SAA, we computed sensitivity values separately for both Aβ DLB groups (Table 4).

**Table 4:** Sensitivity Values Across All 4 Laboratories for Aβ^−^ and Aβ^+^ Group Samples, With or Without Repeating Samples for SAA

Laboratories	Inconclusive SAA results for patients	Aβ^−^ sensitivity (in %)			Aβ^+^ sensitivity (in %)		
		Initial Aβ^−^ sensitivity	Actual Aβ^−^ sensitivity	Final Aβ^−^ sensitivity	Initial Aβ^+^ sensitivity	Actual Aβ^+^ sensitivity	Final Aβ^+^ sensitivity
Lab A	None	**100** (CI 72–100) (10/10) PPV 100 (CI 72–100)	**100** (CI 72–100) (10/10) PPV 100 (CI 72–100)	**100** (CI 72–100) (10/10) PPV 100 (CI 72–100)	**100** (CI 72–100) (10/10) PPV 100 (CI 72–100)	**100** (CI 72–100) (10/10) PPV 100 (CI 72–100)	**100** (CI 72–100) (10/10) PPV 100 (CI 72–100)
Lab B	2 cases (repeated)	**100** (CI 70–100) (9/9) PPV 100 (CI 72–100)	**90** (CI 59–99) (9/10) PPV 100 (CI 70–100)	**100** (CI 72–100) (10/10) PPV 100 (CI 72–100)	**67** (CI 35–88) (6/9) PPV 100 (CI 61–100)	**60** (CI 31–83) (6/10) PPV 100 (CI 61–100)	**70** (CI 40–89) (7/10) PPV 100 (CI 61–100)
Lab C	5 cases (not repeated)	**86** (CI 49–99) (6/7) PPV 100 (CI 61–100)	**60** (CI 31–83) (6/10) PPV 100 (CI 61–100)	**60** (CI 31–83) (6/10) PPV 100 (CI 61–100)	**63** (CI 31–86) (5/8) PPV 100 (CI 57–100)	**50** (CI 24–76) (5/10) PPV 100 (CI 57–100)	**50** (CI 24–76) (5/10) PPV 100 (CI 57–100)
Lab D	2 cases (not repeated)	**100** (CI 70–100) (9/9) PPV 100 (CI 72–100)	**90** (CI 59–99) (9/10) PPV 100 (CI 70–100)	**90** (CI 59–99) (9/10) PPV 100 (CI 70–100)	**67** (CI 35–88) (6/9) PPV 100 (CI 61–100)	**60** (CI 31–83) (6/10) PPV 100 (CI 61–100)	**60** (CI 31–83) (6/10) PPV 100 (CI 61–100)

Abbreviations: Aβ = β-amyloid; PPV = positive predictive value; SAA = seed amplification assay.

Initial sensitivity was calculated after first run of SAA where inconclusive cases were excluded from the analysis. Actual sensitivity was calculated by considering inconclusive cases as failure (nonsuccess), with or without repeating samples for SAA. Final sensitivity was calculated after repeat SAA for inconclusive cases. Lab A did not have any inconclusive cases after initial SAA; therefore, actual and final sensitivity are the same as initial sensitivity. Sensitivity values are given in percentage with 95% CIs. PPVs are shown with 95% CIs. Labs C and D did not repeat inconclusive cases, and therefore, final sensitivity is the same as actual sensitivity. Numbers in bracket show the number of samples, which tested positive or negative out of the total number of samples. Sensitivity values are reported as percentage (bold number).

The initial analysis for Lab A showed a sensitivity of 100% for both Aβ^−^ (10/10) and Aβ^+^ (10/10) patients with DLB, and there were no inconclusive cases; hence, the initial analysis corresponds to the final analysis.

The initial analysis for Lab B showed a sensitivity of 100% (9/9) for Aβ^−^ patients with DLB. The actual sensitivity was 90% (9/10), and the final sensitivity after reanalysis was 100% (10/10). The initial analysis of Aβ^+^ patients with DLB resulted in a sensitivity of 67% (6/9), with an actual sensitivity of 60% (6/10) and a final sensitivity of 70% (7/10).

The initial analysis for Lab C showed a sensitivity of 86% (6/7) for Aβ^−^ patients with DLB, with an actual and final sensitivity of 60% (6/10). For Aβ^+^ patients with DLB, the initial sensitivity was 63% (5/8), with an actual and final sensitivity of 50% (5/10).

The initial analysis for Lab D showed a sensitivity of 100% (9/9) for Aβ^−^ patients with DLB, with an actual and final sensitivity of 90% (9/10). For Aβ^+^ patients with DLB, the initial sensitivity was 67% (6/9), with an actual and final sensitivity of 60% (6/10).

In summary, the sensitivity values were generally higher for Aβ^−^ patients with DLB (average sensitivity = 87.5%) than for Aβ^+^ patients with DLB (average sensitivity = 70%). However, these differences in sensitivity were nonstatistically significant (*p* > 0.05 pairwise intralaboratory).

### Agreement of SAA Results Across Laboratories

Agreement analysis was performed on the final sensitivity and specificity values. Labs A and B showed substantial agreement (κ = 0.714), and both Labs A and B showed fair agreement with Lab D (κ = 0.341). Lab C showed slight agreement with the other 3 laboratories (κ = 0.133). Overall, when averaging all pairwise κ values, we observed fair agreement across laboratories (κ = 0.246) (supplementary file provides full results).

### Comparison of the SAA Protocol Across Laboratories

We qualitatively assessed differences and similarities in SAA protocols across laboratories (eTable 1). All laboratories used recombinant α-syn, either expressed and purified locally (Lab A) or purchased (Labs B, C, and D). All laboratories ran the assay in 96-well plates. The final α-syn concentration varied between Labs A (0.07 mg/mL) and B (0.1 mg/mL), whereas Labs C and D used the same concentration of α-syn (0.3 mg/mL). Labs A and B used 15 μL of CSF per well, whereas Labs C and D used 40 μL of CSF. SAA was run at 42°C in Labs A and C and at 37°C in Labs B and D. Buffer pH varied between Labs A (pH 8) and B (pH 5.5) but was the same for Labs C and D (pH 6.5). Sodium chloride salt concentration in the buffer varied between Labs A (170 mM) and B (no sodium chloride) but was the same for Labs C and D (500 mM).

### Classification of Evidence

This study provides Class III evidence of variations in the accuracy of CSF α-syn SAA across 4 separate laboratories in distinguishing patients with DLB from healthy controls.

## Discussion

We performed a cross-validation of the α-syn SAA using CSF samples from patients with DLB and controls across 4 laboratories. We demonstrated an average final diagnostic accuracy of 78.5%, with variation across laboratories. The α-syn SAA showed lower sensitivity in Aβ-positive patients with DLB (70%) than in Aβ-negative patients (87.5%). Overall, a fair agreement was observed for SAA results across laboratories (κ = 0.246).

Regarding our first specific aim, to compare sensitivity, specificity, and accuracy values of the α-syn SAA across laboratories, Lab A achieved a perfect diagnostic accuracy (100% sensitivity and specificity), consistent with a previous study achieving 97% sensitivity using a similar SAA method.^19^ However, our study revealed variation in sensitivity and specificity values across laboratories. Specifically, Lab B showed 87% diagnostic accuracy, with 3 patients with DLB testing negative and 1 control testing positive. Of interest, these 3 patients with DLB were also negative or inconclusive in Labs C and D, and all 3 patients were Aβ^+^. This concordance across Labs B, C, and D may indicate a lower amount of misfolded α-syn in these 3 patients with DLB than in the other 17 patients with DLB or Aβ interacting with α-syn aggregation,^20^ perhaps affecting the SAA result. Labs C and D showed substantially lower accuracy values (57% and 70%, respectively), on final analysis. One reason for this may be their inability to repeat SAA for inconclusive cases. Diagnostic accuracy improved for Labs A and B after repeating initial SAA analysis, which supports this hypothesis. Other possible reasons could be different fluorescence cutoff points and assay time in Labs C and D.

Our study included 2 patients with DLB with a neuropathologic confirmation of Lewy body disease. These 2 patients with DLB were SAA positive (100%, 2/2) in Labs A and B, while Labs C and D showed inconclusive results for 1 patient each. This suggests that the SAA reliably detects α-syn pathology in CSF, but the result is not fully reproducible across laboratories. This finding is in line with 2 single-center publications reporting that SAA is not always positive in neuropathologically confirmed patients, including those with sDLB (93% SAA positive in Ref. ^21^ and 98% in Ref. ^22^).

Our second aim was to study the effect of Aβ status. This informed the potential influence of Aβ co-pathology on SAA results in DLB, in contrast to previous multilaboratory studies that did not report such data.^14,15,23^ We observed lower sensitivity values for Aβ^+^ patients with DLB (70%) than Aβ^−^ patients with DLB (87.5%). Despite these qualitative differences, the small number of patients per Aβ group (n = 10) may have limited the statistical power to detect significant differences. The different sensitivities could be explained by cross-seeding of Aβ with α-syn in SAA.^20,24^ Understanding this interaction is vital, and an urgent prospect for future DLB studies is to confirm the impact of an Aβ-positive biomarker status on SAA results. This may have important diagnostic implications because around 50% of patients with DLB are positive for Aβ biomarkers.^5^ If future studies confirm that Aβ positivity affects SAA results, it is then crucial to develop SAA protocols that are robust to Aβ co-pathology.

Our third aim was to analyze the agreement of SAA results across laboratories. Only 3 previous reports compared the α-syn SAA across multiple laboratories. One study included 2 laboratories with 34 patients with PD and 30 controls, using skin biopsies.^23^ The authors showed almost perfect agreement between the 2 laboratories (κ = 0.861).^23^ However, both laboratories used the same SAA protocol with only slight modifications. A strength of our study is that the α-syn SAA was run across 4 independent laboratories, each using a different protocol. This scenario likely reflects more closely the real-world performance of SAA in the clinical setting. In this context, our study showed that the agreement is lower, with the κ value of 0.861 reported in Ref. ^23^ dropping to an average κ value of 0.246 in our study. A recent 4-laboratory comparison of α-syn SAA in CSF, including 14 patients with PD, 4 patients with DLB, and 20 controls, showed a similar κ value (0.280).^15^ The third published study is a 3-laboratory comparison of α-syn SAA in CSF, including 30 patients with PD and 30 controls.^14^ κ Values were not reported in that study, while the authors showed a high sensitivity of 86%–96% and specificity of 97%–100%,^14^ which probably corresponds to a moderate-to-substantial agreement.

The final aim of our study was to assess differences and similarities of the SAA protocol across laboratories. Laboratories used α-syn either obtained from a commercial source or produced in a protein production facility. A previous publication showed that α-syn protein purification grade and use of purification tags such as His-tag affect the SAA.^12^ We also observed variation in buffer conditions including salt, pH, and SDS content. These factors are known to influence protein aggregation kinetics,^25^ and hence, they will also affect SAA performance, likely contributing to variation in SAA performance across our 4 laboratories.

Previous studies have shown that the SAA results are also dependent on fluorescence cutoff thresholds and assay time duration.^12,26^ Lab A used a cutoff for ThT fluorescence, but there was no cutoff for assay time. By contrast, Labs B and D used cutoff values for both ThT fluorescence and time. Notably, Lab D used a median fluorescence cutoff of 3 replicates for CSF samples tested positive or negative for SAA, whereas the other laboratories used cutoff fluorescence for each replicate rather than using the median of all replicates. The CSF samples were considered negative if the ThT fluorescence of all replicates was below the ThT cutoff fluorescence. The sample was inconclusive if only 1 replicate was positive. The number of replicates across laboratories also varied with either 4 (Lab A) or 3 (for the other 3 laboratories) replicates. This higher number of replicates in Lab A may have contributed to its improved accuracy by reducing the impact of variability or borderline results. Our study thus shows that there is an urgent need to harmonize cutoff criteria for fluorescence, number of replicates, and time of assay for the interpretation of SAA results in clinical routine. Because these factors have a major impact in accurately diagnosing people with and without the disease, such harmonization should be a priority for the clinical implementation of the α-syn SAA.

Two recent reviews discussed other factors that should be considered for harmonization such as reagents and protocols.^10,12^ Furthermore, a known limitation of the α-syn SAA is its current qualitative results (i.e., positive vs negative), with the lack of a robust way to quantify and interpret results. Our study demonstrates 2 additional limitations of SAA for its clinical implementation: the lack of standardization of the SAA protocol and currently just fair reproducibility across laboratories. Our report pointing at technical variation in α-syn preparation, experimental conditions, and analysis of SAA results may elucidate several areas for improvement in future studies.

Our study has some limitations. First, the number of patients was twice the number of controls, which might slightly favor the accuracy of sensitivity values in comparison with the specificity values. Second, the SAA could not be repeated in Labs C and D for inconclusive cases because of insufficient amount of CSF. Labs C and D required higher amounts of CSF for the assay, while equal amounts of CSF were available to all laboratories by study design. Sensitivity and specificity values of Labs C and D might have improved if the SAA could be repeated for inconclusive cases. However, the initial SAA results offer some insights, showing that the accuracy was still higher for Labs A, B, and D (100%, 87%, and 84%, respectively) than for Lab C (77%). Third, we had a neuropathologic confirmation for only 2 patients with DLB. While the lack of postmortem confirmation is a common issue in any biomarker study of DLB, we minimized this by increasing diagnostic certainty through requirement of a positive DaT-SCAN^27^ by study design. Still, the gold standard in this study was the clinical diagnosis, and we cannot exclude that some patients with DLB may not have α-syn pathology in this cohort. For example, patients 14, 16, and 19 were consistently labeled as negative by Labs B, C, and D, and this could indicate that Lab A may have labeled them as false positives. However, we carefully inspected the clinical records of these 3 patients, and their clinical picture was fully compliant with typical DLB for patients 16 and 19, and patient 14 was clinically diagnosed with DLB with a positive DaT-SCAN. Therefore, neuropathologic confirmation for all patients with DLB is required to provide a definitive assessment of the performance of the α-syn SAA across laboratories. Fourth, although the α-syn SAA has a high performance in discriminating DLB from AD,^11^ we did not include a group of patients with a clinical diagnosis of AD, which would have further informed the clinical utility and implementation of the α-syn SAA.

In conclusion, the α-syn SAA in CSF has the potential to be implemented clinically for the biomarker-based diagnosis of DLB (and PD). However, the reported variability in sensitivity and specificity values across our 4 laboratories and fair agreement suggest that the next step is to propose international recommendations for the harmonization of the α-syn SAA for clinical use. Such recommendations should encourage the use of an affordable, readily, and consistently available recombinant substrate with high availability, establish the criteria for determining a positive SAA result, propose standards for the harmonization of SAA protocols, provide an international quality assurance scheme, and recommend when to test patients.

## Glossary

α-syn: α-synuclein
Aβ: β-amyloid
AD: Alzheimer disease
DaT: dopamine transporter
DLB: dementia with Lewy bodies
MMSE: Mini-Mental State Examination
PD: Parkinson disease
RBD: REM sleep behavior disorder
SAA: seed amplification assay
ThT: Thioflavin T
VH: visual hallucination
